# Editorial: Social Inequities in Cancer

**DOI:** 10.3389/fonc.2019.00233

**Published:** 2019-04-04

**Authors:** Dana Hashim, Friederike Erdmann, Hajo Zeeb

**Affiliations:** ^1^Department of Hematology and Oncology, Icahn School of Medicine at Mount Sinai, Tisch Cancer Institute, New York, NY, United States; ^2^Childhood Cancer Research Group, Danish Cancer Society Research Center, Copenhagen, Denmark; ^3^Section of Environment and Radiation, International Agency for Research on Cancer, Lyon, France; ^4^Department of Prevention and Evaluation, Leibniz-Institute for Prevention Research and Epidemiology-BIPS GmbH, Bremen, Germany; ^5^Health Sciences Bremen, University of Bremen, Bremen, Germany

**Keywords:** social inequalities, socioeconomic status, global health, social inequities, cancer epidemiology, cancer disparities, social epidemiology

Social inequalities and equities are very closely related, but with important differences. In cancer epidemiology, social inequalities refer to differences in socioeconomic position (SEP) related to statistical differences in incidence, mortality, and survival rates between populations. Social inequities may be the cause of social inequalities. Social inequities are systemic, unnecessary, unjust, and avoidable barriers that prevent segments of the population from achieving optimal health (1). Geographical, economic, societal, and cultural aspects of inequity interact to construct circumstances in which these subgroups are, to varying degrees, excluded or included. As populations navigate the cancer care continuum of cancer prevention, detection/diagnosis, and management/treatment (2), ingrained social inequities lead to cancer incidence, mortality, and/or survival disparities (3–5). Social inequities have been recognized in numerous studies as a strong predictor of morbidity and premature mortality worldwide (6) and contribute to cancer inequalities within countries and between countries (7). Although reductions in cancer burden are achievable by reducing social and economic inequities, socioeconomic factors and their role in cancer causation and outcomes are often not targeted in public health strategies.

The field of “social epidemiology” is distinguished by its focus on the conditions of the environment in which population subgroups grow, work, and live, encompassing the cumulative impact of these factors—the social determinants—, as a whole, on health, and disease outcomes (7, 8). The study of social inequities in cancer prevention strategies is a field of active research, e.g., with a recent publication identifying low social class based on occupational title as having a positive relationship with cancer mortality (4) as well as the recent incorporation of a socio-demographic index (SDI) to annual Global Burden of Disease reporting to stratify disease burden (9–11).

It must be acknowledged that targeting social inequities to improve public health requires attention to concepts and methods conducive to illuminating links between our physiology and social, political, and economic systems (12). Several studies in this current topical issue focus on analyses of cancer incidence, mortality, and survival by measures of socioeconomic status using Baysian models, area-based socioeconomic indices (Carstairs, Theil T), human development index (HDI), and a childhood/adolescent SEP based on parents' ownership of a car. The goal of this research topic is to draw attention to several aspects of social inequities, including identifying unequal distributions of cancer in social groups, health care system research, specific risks among less-studied ethnic groups including life course models, and cancer survival inequities.

## Unequal Distribution of Cancer in Social Groups

Kamath et al. provide an in-depth account of social disparities in liver cancer frequency, risk factors as well as preventive services in New York City, known for its mixed ethnic and social composition. Their study is an excellent example of using multiple existing data sources in order to shed light on cancer related-disparities at neighborhood level, with concomitant illustration using geographical mapping.

Germany has a large immigrant population, established since the 1960s and recently expanded in the wake of large refugee movements. The “Aussiedler” (resettlers) are a unique population consisting of ethnic Germans formerly residing in the ex-USSR. Kaucher et al. report on two large administrative data-based-cohorts and show that initially elevated frequencies of stomach and lung cancer (among men) converge to the risk among the majority population, whereas mortality remains largely unchanged. Analyses of colorectal, prostate, and female breast cancer incidence rates reveal patterns favoring the migrant population. Unfortunately, there are no data on relevant life-style and other risk factors in this study and ethnicity was used as a proxy for SEP.

Using an area-based measure of social deprivation, Hoebel et al. study the socially unequal distribution of cancer risk in Germany. They largely confirm international results, also in terms of reverse gradients for malignant melanoma, breast and thyroid cancer. Their analysis provides insights into both absolute and relative inequalities and indicate that overall, there are larger social inequalities in cancer among men compared to women. However, site-specific analyses differentiate this picture to some extent.

Cervical cancer remains at the top of important cancers for many less developed countries. Santamaría-Ulloa and Valverde-Manzanares provide an account of existing social differences in cervical cancer incidence in Costa Rica. The economic dimension of the index used is a compound measure of residential electricity consumption and residential access to internet and the Theil T index used to quantify inequality on a district level. Higher incidence rates are found to be related to a lower uptake of cervical cancer screening, and rates differ substantially across socioeconomic regions within Costa Rica.

On the global level, Fidler and Bray use the HDI as composite metric to study global cancer frequencies. They outline HDI stratification as an important approach providing guidance for the development and implementation of cancer control plans worldwide. A notable characteristic of the HDI is the fact that it combines social (education), health (life expectancy), and economic (gross national income) data at country level. Further discussion is warranted regarding how the HDI compares to the SDI used in the Global Burden of Disease studies.

## Life Course, Genetic-Ethnic Issues

Little is known about prostate cancer risk factors, although blacks have a much higher rate than whites. Madathil et al. investigate the relationship between lifelong SEP and prostate cancer in a French-speaking Canadian population using a Bayesian life course exposure model. Measures of SEP during childhood/adolescence include parents' ownership of a car and father's longest occupation, while the subject's first and longest occupations indicate early- and late-adulthood SEP. Lower SEP over the life course is associated with higher PCa incidence, with evidence for sensitive time periods.

Brovkina et al. focus on hereditary breast and ovarian cancer syndrome (HBOS) among Tatars, one of the largest ethnic minority groups in Russia. It was previously reported that the BRCA mutation, while frequent for the Slavic population, has not been found in Tatar women with hereditary breast cancer. This study demonstrates a predisposition for the C*DK12*c.1047-2A>G nucleotide variant in HBOCS in patients of Tatar ethnicity and identifies C*DK12* as a novel gene involved in HBOCS susceptibility.

## Health Care Systems and Cancer Research

The study by Alavi et al. is the first to focus on public versus private rehabilitation centers in Iran. Private rehabilitation centers were rated higher in communication, basic amenities and autonomy compared to public centers. Using the Blinder-Oaxaca decomposition model, perceived social class explain 76% of the inequality in autonomy in choosing between public and private rehabilitation center.

With a broad perspective on potentials for cancer research, the review by Drake et al. outlines the methods by which funding schemes, scientists, genome consortia, and policy makers can play a role to ensure cancer research is generalizable and beneficial to patients in both high- and low-income countries. This includes higher representation of low-to-middle income countries in large molecular and genomic studies, focus on cost-effective approaches to precision medicine, and an overall pooling of data and resources to foster the mechanistic understanding of cancer on a global level.

## Survival and Social Factors

Survival rates have substantially improved over the last decades for most cancer sites. Nonetheless, not all patients benefit from these advances. It has been consistently observed that socioeconomically disadvantaged cancer patients have worse survival than patients from socioeconomically advantaged groups and, in some countries, this socioeconomic gap has widened over time.

Ingarfield et al. assess the change in social inequality in the survival of patients with head and neck cancer between short-, mid-, and long-term survival in Scotland. Findings show a clear gradients in overall, disease-specific and net survival across socioeconomic groups (measured by area-based Carstairs 2001 index). Further analyses with full adjustment reveal that the survival inequalities can be largely explained by differences in multiple factors, including patient, tumor, and treatment.

Finke et al. conduct a systematic review and meta-analysis synthesizing current knowledge on socioeconomic differences in lung cancer survival with a particular focus on differences by measurements of socioeconomic status used (individual-level vs. ecological grouping). Findings from the meta-analyses indicate a poorer prognosis among lower income patients. While no evidence for associations between individual education or occupation and lung cancer survival are observed, studies using an area-based socioeconomic measure show lower survival for lower socioeconomic groups. Of note, only eight of the 94 reviewed individual studies account for smoking status in their analysis.

Evidence is accumulating that for childhood cancer, socioeconomic and social factors also impact survival. Mogensen et al. review the most recent publications on social and socioeconomic factors and childhood cancer survival in high-income countries and find the evidence to be heterogeneous. Some studies observe no survival differences between children by socioeconomic background, while several studies indicated a social gradient with higher mortality among children from families of lower SES. Mogensen et al. note that knowledge on underlying mechanisms for social inequalities in survival is lacking.

Social inequities affect all aspects of cancer, from research to health care systems, from disparities in incidence to treatment outcome, and life after cancer. It is also a topic that has recently become high priority with the increasing burden of cancer worldwide. As a result of improving survival rates (13), the number of cancer survivors is continuously increasing. Access to health information and globalization are also introducing a wider range of social groups to screening, diagnostic, and treatment services as well as exposing disparities in access to health services. The public health relevance of social inequities is substantially increasing and will continue to be an important consideration to explain observed differences in cancer incidence, mortality, and survivorship—even in the near future.

While the studies presented in this twelve-article collection cannot comprehensively cover a topic of expanding breadth and depth, the new research questions raised in the individual articles highlight the knowledge gaps, socioeconomic metrics, and analytical techniques on the subject of social inequities. In doing so, this collection contributes to identifying opportunities in reducing social inequality gaps and, therefore, overall cancer burden, by providing an evidence-based foundation to build on public health research aimed at reducing the social inequity in cancer.

## Author Contributions

DH conceived the draft and wrote the manuscript. FE and HZ contributed to the manuscript text and editing. HZ supervised the manuscript writing process. All authors provided critical feedback and helped shape the direction of the manuscript.

### Conflict of Interest Statement

The authors declare that the research was conducted in the absence of any commercial or financial relationships that could be construed as a potential conflict of interest.
